# Combined exercise and dietary interventions for body composition in adults with overweight or obesity: a comparator-stratified systematic review and meta-analysis of randomized trials

**DOI:** 10.3389/fnut.2026.1969222

**Published:** 2026-10-28

**Authors:** Qiang Li, Zhixing Chen, Gang Xu, James J. Zhang

**Affiliations:** 1 School of Physical Education, Shangrao Normal University, Shangrao, China; 2 School of Physical Education, Huizhou University, Huizhou, China; 3 School of Physical Education, Sichuan University of Science and Engineering, Zigong, China; 4 Department of Kinesiology, University of Georgia, Athens, GA, United States

**Keywords:** body composition, dietary, exercise, meta-analysis, overweight or obese, systematic review

## Abstract

**Objective:**

This study aimed to evaluate the effects of combined exercise-dietary interventions on body weight (BW), body mass index (BMI), fat-free mass (FFM), and fat mass percentage (FM%) in adults with overweight or obesity compared with randomized comparators.

**Methods:**

PubMed, Embase, Web of Science, and the Cochrane Library were searched on 28 September 2026. Twenty-three reports representing 21 unique parent trials were included. Post-intervention arm data were synthesized as comparator-specific mean differences using restricted maximum likelihood random-effects models with truncated Hartung–Knapp confidence intervals, Tau-squared, and 95% prediction intervals. Cross-comparator and hierarchical parent-trial models were used as sensitivity analyses. Risk of bias was assessed using the revised Cochrane Risk of Bias tool for randomized trials (RoB 2), and certainty of evidence was evaluated using the Grading of Recommendations Assessment, Development, and Evaluation (GRADE) approach.

**Results:**

The BW analysis included 1,303 participant observations. Against dietary-only controls, combined interventions reduced BW by −4.49 kg (95% confidence interval −8.05 to −0.92), and reduced BMI by −1.50 kg m^−2^ (−2.67 to −0.34), but both prediction intervals crossed the null. Exercise-only comparisons were imprecise, while active dietary and exercise comparisons and minimal guidance comparisons each relied on one report. No comparator stratum showed reliable improvement in FFM or FM%. Cross-comparator sensitivity estimates favored combined interventions for BW and BMI, but every prediction interval crossed the null. Certainty of the estimates was very low for all outcomes.

**Conclusion:**

Combined interventions may reduce BW and BMI compared with trial-specific comparators in comparisons with dietary-only controls, but heterogeneity and sparse strata limit certainty. The evidence did not establish FFM preservation, the superiority of one dietary intervention type, or clinical importance.

**Systematic review registration:**

https://www.crd.york.ac.uk/PROSPERO/, identifer CRD420251174901.

## 1 Introduction

Obesity is a major global public health challenge associated with cardiovascular disease, type 2 diabetes, several cancers, disability, and substantial healthcare costs (1–5). Its persistence reflects interacting biological, behavioral, environmental, and socioeconomic determinants rather than a single modifiable exposure. Thus, management commonly requires coordinated dietary and physical activity strategies.

Dietary energy restriction and structured physical activity are core components of obesity management (6–10). Either component alone can be limited by compensatory changes in appetite, energy expenditure, adherence, or physical activity. Combining diet and exercise can increase the energy deficit while supporting cardiorespiratory fitness, strength, and functional capacity; the incremental effect of a combined program therefore depends on the specific intervention and its comparison group.

Dietary strategies represented in combined programs are not physiologically or methodologically interchangeable. Continuous caloric restriction (CR) directly targets energy intake, whereas time-restricted eating or fasting (TRF), intermittent fasting, alternate-day fasting, and Ramadan fasting primarily alter eating timing or frequency and may not create a comparable energy deficit (11, 12). These strategies must therefore remain distinguishable in classification and interpretation.

Exercise exposures also differ. Aerobic, resistance, concurrent, walking, and interval protocols vary in energy expenditure, mechanical loading, dose, supervision, and expected effects on body composition (13). Adherence, protein intake, intervention duration, baseline adiposity, and the measurement method may further modify the observed effects.

Previous syntheses have compared broad exercise and dietary combinations, direct component contrasts, or specific clinical populations (14, 15). Recent reviews have also emphasized variations in populations, interventions, and comparators (16, 17). These reviews address related questions but do not resolve the meaning of pooled estimates across different randomized comparators.

A combined program compared with diet alone estimates the added contribution of exercise. The same program compared with exercise alone estimates the added contribution of the dietary prescription. A comparison with minimal guidance estimates the total package effect. An active alternative diet and exercise comparator addresses a different contrast. Pooling these comparisons can provide a broad sensitivity summary, but it does not estimate an effect against one common standard control.

This review therefore prioritizes comparator-specific estimates, uses differences in natural units, maps reports to unique parent trials, distinguishes participant observations from unique participants, and treats dietary intervention-type analyses as exploratory rather than direct comparisons. This study aimed to estimate effects on BW, BMI, FFM, and FM%; explore heterogeneity; and assess risk of bias, certainty, and clinical interpretability.

## 2 Materials and methods

### 2.1 Protocol and reporting

This systematic review and meta-analysis was reported in accordance with the Preferred Reporting Items for Systematic Reviews and Meta-Analyses (PRISMA) 2020 statement and was informed by the Cochrane Handbook for Systematic Reviews of Interventions (18).

The protocol was prospectively registered with the International Prospective Register of Systematic Reviews (PROSPERO) (CRD420251174901). Comparator stratification, natural unit reanalysis, parent trial mapping, prediction intervals, meta-regression analyses, and the GRADE assessment were added after protocol registration and were interpreted with appropriate caution.

### 2.2 Search strategy and study selection

Two reviewers independently searched PubMed, Embase, the Cochrane Library, and Web of Science from database inception to 28 September 2026.

Citation searching identified three additional records. The searches combined controlled vocabulary and free text for the terms “overweight” or “obesity,” “exercise,” “dietary intervention,” and “randomized trials.” The complete executable strategies and archived counts are reported in Supplementary Table S1.

The searches retrieved 2,323 records from PubMed, 5,648 from Embase, 3,592 from Cochrane Central Register of Controlled Trials, and 4,965 from Web of Science. The search results were exported and deduplicated before screening. No language restrictions were applied at the search stage. Two reviewers (ZC and GX) independently screened titles, abstracts, and full-text articles using EndNote 21; disagreements were resolved through discussion or consultation with a third reviewer (QL).

### 2.3 Inclusion and exclusion criteria

Eligibility was determined using the prespecified Population, Intervention, Comparison, Outcome, and Study (PICOS) criteria:

1. Population: adults aged 18 years or older with overweight or obesity.
2. Intervention: structured exercise combined with a dietary strategy, including CR, intermittent fasting, TRF, alternate-day fasting, or a low-carbohydrate diet.
3. Comparator and estimand: eligible comparators included dietary-only controls, exercise-only controls, and no-intervention controls (delayed physical activity, normal eating, or usual lifestyle). The overall cross-comparator estimate therefore represents the mean effect of assigning participants to a combined exercise-diet program rather than to the diverse comparators selected by each trial; it does not represent a common standard of care or a direct comparison between combined strategies. Comparator-stratified estimates were treated as the primary basis for interpretation.
4. Outcomes: usable quantitative data for BW, BMI, FFM, or FM%; and
5. Design: randomized parallel-group or crossover trials. For crossover trials, paired estimates or first-period data were required to avoid unit-of-analysis errors.

The exclusion criteria were as follows:

1. Non-human or non-randomized studies;
2. Interventions that did not include both exercise and a dietary component;
3. Insufficient quantitative data to estimate an effect; and
4. Conference abstracts, reviews, protocols, duplicate reports, or reports without an eligible comparator.

### 2.4 Data extraction and comparator classification

Two reviewers independently re-extracted arm-level sample sizes, postintervention means, and standard deviations using a standardized form. The extracted descriptors included publication details, population, randomized arms, dietary prescription, exercise modality and dose, supervision, adherence, body composition method, and analysis time point. Discrepancies were resolved by consensus, with adjudication by a third reviewer when required. Study and intervention characteristics are reported in Supplementary Table S3.

All analyses used postintervention endpoint values. Change scores were not mixed with endpoints. One prespecified contrast was entered per report and outcome. When a report contained several eligible arms, the contrast was selected in the following order: the combined arm against the same diet without added exercise, the combined arm against the same exercise without the experimental diet, an active alternative diet and exercise arm, and finally minimal guidance. A shared comparator was entered once within each report.

A report was defined as a publication contributing an eligible contrast, a unique parent trial was defined as an independently randomized cohort, and a participant observation was defined as a report-specific contribution to an outcome analysis. Because participants could recur in companion reports, participant observations were not interpreted as unique persons across reports. Report identifiers were standardized by the first author and publication year. The full mapping of reports to references is provided in Supplementary Table S3.

Among the 23 included reports, Goodpaster (2010) and Cooper (2012) were mapped to one unique parent trial, and Brinkley (2011) and Wang (2015) were mapped to NCT00664729, yielding 21 unique parent trials. All 23 reports were retained because companion reports contributed eligible, report-specific outcome data, and selecting a report only based on publication date would have discarded relevant data. One prespecified contrast per report and outcome limited duplication within reports. The primary, comparator-specific analyses used report-level estimates, and their interpretation was required to be consistent with the results of a prespecified, hierarchical dependency analysis with a random intercept for the parent trial. Exact within-trial sampling correlations were unavailable, so residual dependence was acknowledged when interpreting certainty. The complete mapping of reports to parent trials is provided in Supplementary Table S3.

Dietary intervention types were recorded as continuous CR, alternate-day fasting, TRF, or Ramadan fasting. These categories were not collapsed into a single fasting class for subgroup analysis. Region, duration, and mean study age were evaluated only as exploratory study-level moderators.

The full arm-level dataset, comparator classifications, parent trial mapping, effect size calculations, Stata code, and analysis log are included in this study.

### 2.5 Risk of Bias and certainty of evidence

Risk of bias for the prespecified body composition outcomes was assessed using the Cochrane RoB 2 tool (version 22 August 2019) to assess the effect of assignment to the intervention. The assessment covered five domains: bias arising from the randomization process; bias due to deviations from the intended interventions; bias due to missing outcome data; bias in the measurement of the outcome; and bias in the selection of the reported result (19). Judgments were categorized as low risk, some concerns, or high risk. Insufficient information for a signaling question was judged as some concerns. The lack of participant blinding was not automatically rated as high risk because the outcomes were measured objectively; the assessment instead considered whether knowledge of the assignment plausibly affected the deviations, missingness, measurement, or selective reporting.

Study-level and outcome-specific judgments with concise rationales are reported in Supplementary Table S2. Certainty was assessed using the GRADE approach for each outcome and comparator contrast (20). Downgrading was considered for risk of bias, inconsistency, indirectness, imprecision, and reporting bias, with explicit reasons shown in Supplementary Table S4.

### 2.6 Statistical analysis

For each outcome, the effect measure was the mean difference, calculated as the combined intervention endpoint minus the comparator endpoint. Negative values indicate lower BW, BMI, or FM% in the combined group. In terms of FFM, positive values favor the combined intervention, while negative values indicate lower FFM.

Comparator-specific analyses were the primary inferential models. Between-study variance, expressed as Tau-squared, was estimated using restricted maximum likelihood. The confidence intervals used the truncated Hartung–Knapp adjustment, which constrains the variance scale to at least one and avoids spuriously narrow intervals in sparse strata (21). A category represented by one report was presented as a single-report estimate rather than a pooled effect.

For strata with at least three reports, 95% prediction intervals were calculated to describe the range of effects expected across similar settings (22). Heterogeneity was summarized with Tau-squared and I-squared. The comparator category was tested as a moderator using a mixed-effects meta-regression analysis. Duration and mean age were exploratory continuous moderators, while dietary intervention type, region, duration category, and age category were displayed only as study-level subgroup analyses. The numerical moderator results are reported in Supplementary Table S5.

An overall, cross-comparator, restricted maximum likelihood model with a truncated Hartung–Knapp adjustment was used only as a sensitivity summary across trial-specific contrasts. Because two unique parent trials contributed companion reports, a prespecified hierarchical random-effects model with reports nested within parent trials was used as a dependency-adjusted companion to the primary analyses. This model retained all report-specific outcomes while evaluating whether the conclusions were sensitive to shared parent trial membership. Leave-one-out analyses assessed influence. Funnel plots and Egger’s tests assessed small-study effects when at least 10 reports were available. A non-significant test result was not interpreted as evidence that reporting bias was absent. Analyses and figures were produced using Stata 18.0 (StataCorp LLC, College Station, TX, United States).

## 3 Results

### 3.1 Search results and study characteristics

The study-selection process is shown in the PRISMA 2020 flow diagram (Figure 1). The four databases yielded 16,528 records, and citation searching identified 3 additional records; the 16,531 records were combined before deduplication. After 11,652 duplicates were removed, 4,879 titles and abstracts were screened; 4,684 records were excluded. A total of 195 full-text reports were assessed for eligibility.

**Figure 1:**
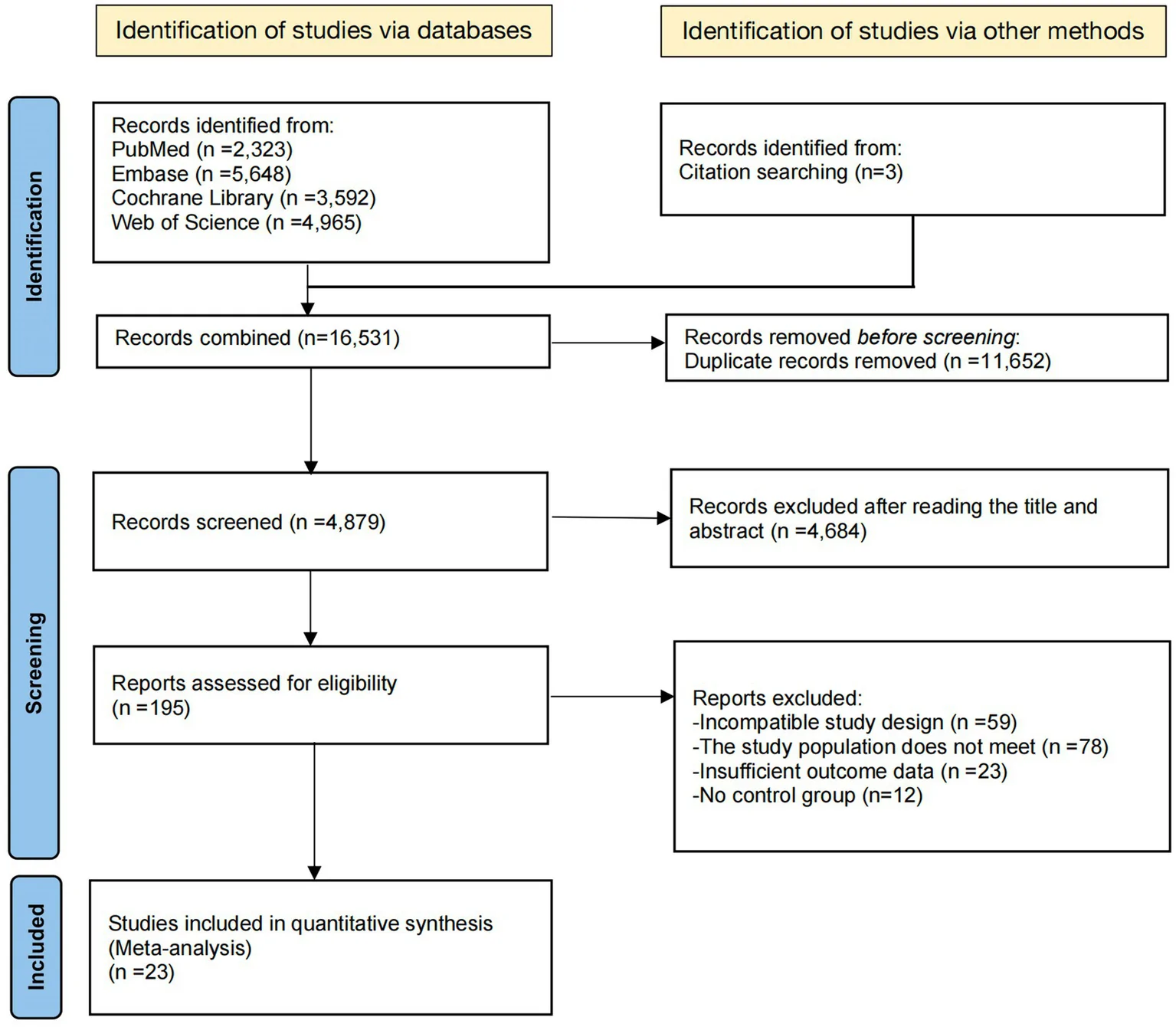
PRISMA 2020 flow diagram of study identification, screening, eligibility, and inclusion. Twenty-three reports representing 21 unique parent trials were included in the quantitative synthesis.

Of these, 172 were excluded because of an ineligible design (*n* = 59), an ineligible population (*n* = 78), insufficient data (*n* = 23), or absence of an eligible comparator (*n* = 12). Twenty-three reports representing 21 unique parent trials were included in the quantitative synthesis. The 23 reports (23–45) contributed 1,303 participant observations to the BW analysis. The outcome-specific totals were 1,145 observations for BMI, 845 for FFM, and 602 for FM%. Because two report pairs resulted from shared parent trials, these totals represent report-level observations rather than unique participants across the entire evidence base.

### 3.2 Risk of Bias and certainty

RoB 2 judgments were predominantly classified as some concerns. Common reasons were incomplete reporting of allocation concealment, open-label lifestyle delivery with limited information on deviations and adherence, incomplete attrition details, and unavailable prespecified analysis plans. BW and BMI measurements generally raised fewer concerns than body composition measurements, which could vary depending on the device used, hydration level, assessor procedures, and analysis choices.

GRADE certainty was very low for every outcome. The principal reasons were risk of bias, serious or very serious inconsistency, imprecision in the comparator strata, and indirectness when a broad cross-comparator summary was considered. Detailed judgments are provided in Supplementary Table S4.

### 3.3 Meta-analysis

#### 3.3.1 Effect on BW

When compared against dietary-only controls, 16 reports yielded a mean difference of −4.49 kg (95% confidence interval −8.05 to −0.92), a Tau-squared value of 30.31, an I-squared value of 90.11%, and a 95% prediction interval of −16.83 to 7.85 kg. When compared against exercise-only controls, five reports yielded a mean difference of −3.42 kg (−9.50 to 2.66), a Tau-squared value of 15.37, an I-squared value of 75.71%, and a prediction interval of −17.71 to 10.87 kg.

One report combining active dietary and exercise interventions yielded a mean difference of −5.10 kg (−14.94 to 4.74), and one report providing minimal guidance yielded a mean difference of −1.20 kg (−10.42 to 8.02). Prediction intervals were not estimable for single-report categories. The test for differences across comparator categories was not significant (*p* = 0.906; Figure 2).

**Figure 2:**
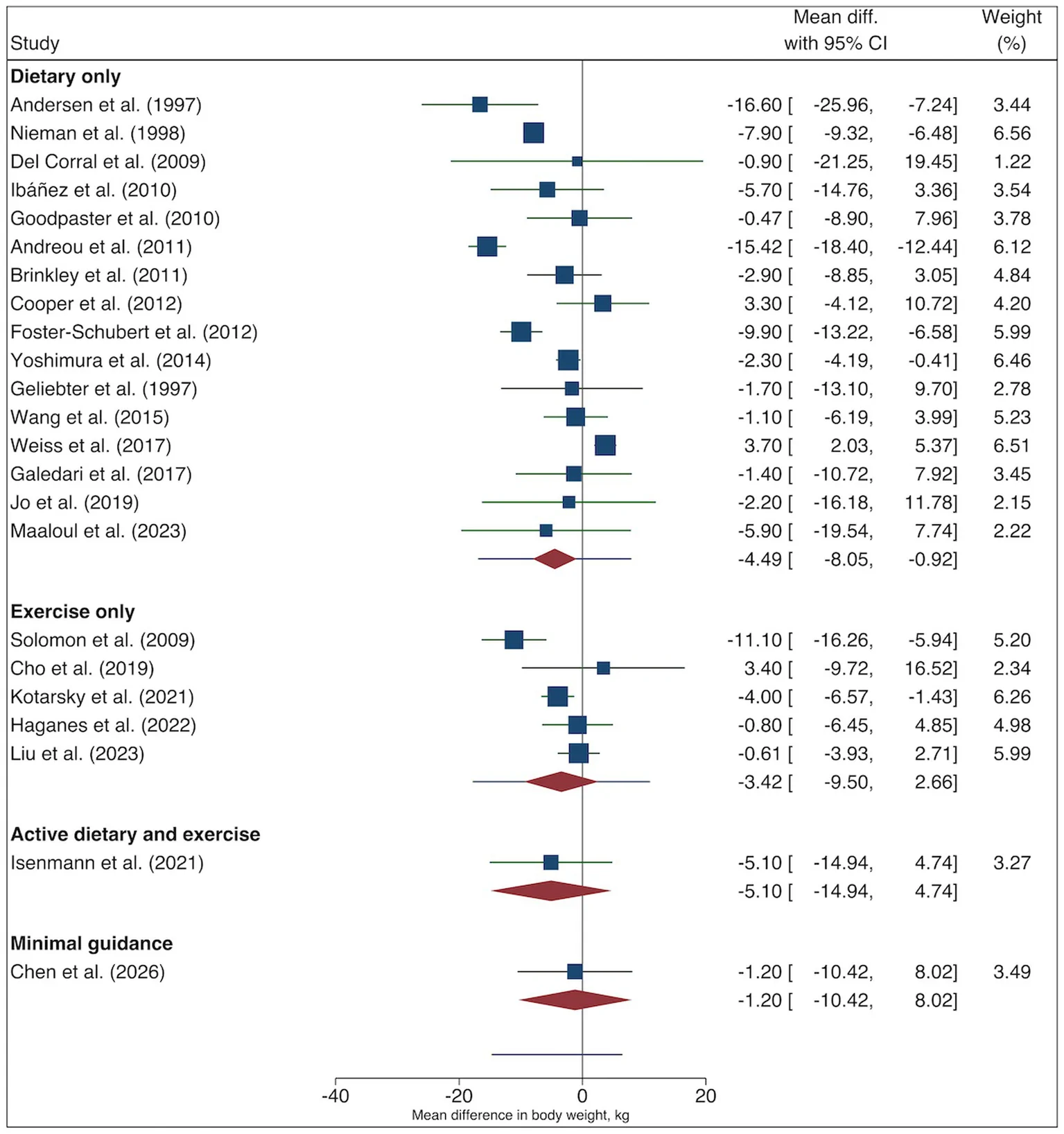
Comparator-stratified random-effects meta-analysis of BW.

#### 3.3.2 Effect on BMI

When compared against dietary-only controls, 12 reports yielded a mean difference of −1.50 kg m^−2^ (95% confidence interval −2.67 to −0.34), a Tau-squared value of 2.35, an I-squared value of 87.29%, and a prediction interval of −5.12 to 2.11 kg m^−2^. When compared against exercise-only controls, four reports yielded a mean difference of −0.97 kg m^−2^ (−5.28 to 3.35), a Tau-squared value of 6.58, an I-squared value of 94.93%, and a prediction interval of −13.45 to 11.52 kg m^−2^.

Single-report estimates were −0.60 kg m^−2^ (−2.69 to 1.49) for the active dietary and exercise comparator and −0.80 kg m^−2^ (−3.07 to 1.47) for the minimal guidance report. The comparator category did not explain heterogeneity (*p* = 0.851; Figure 3).

**Figure 3:**
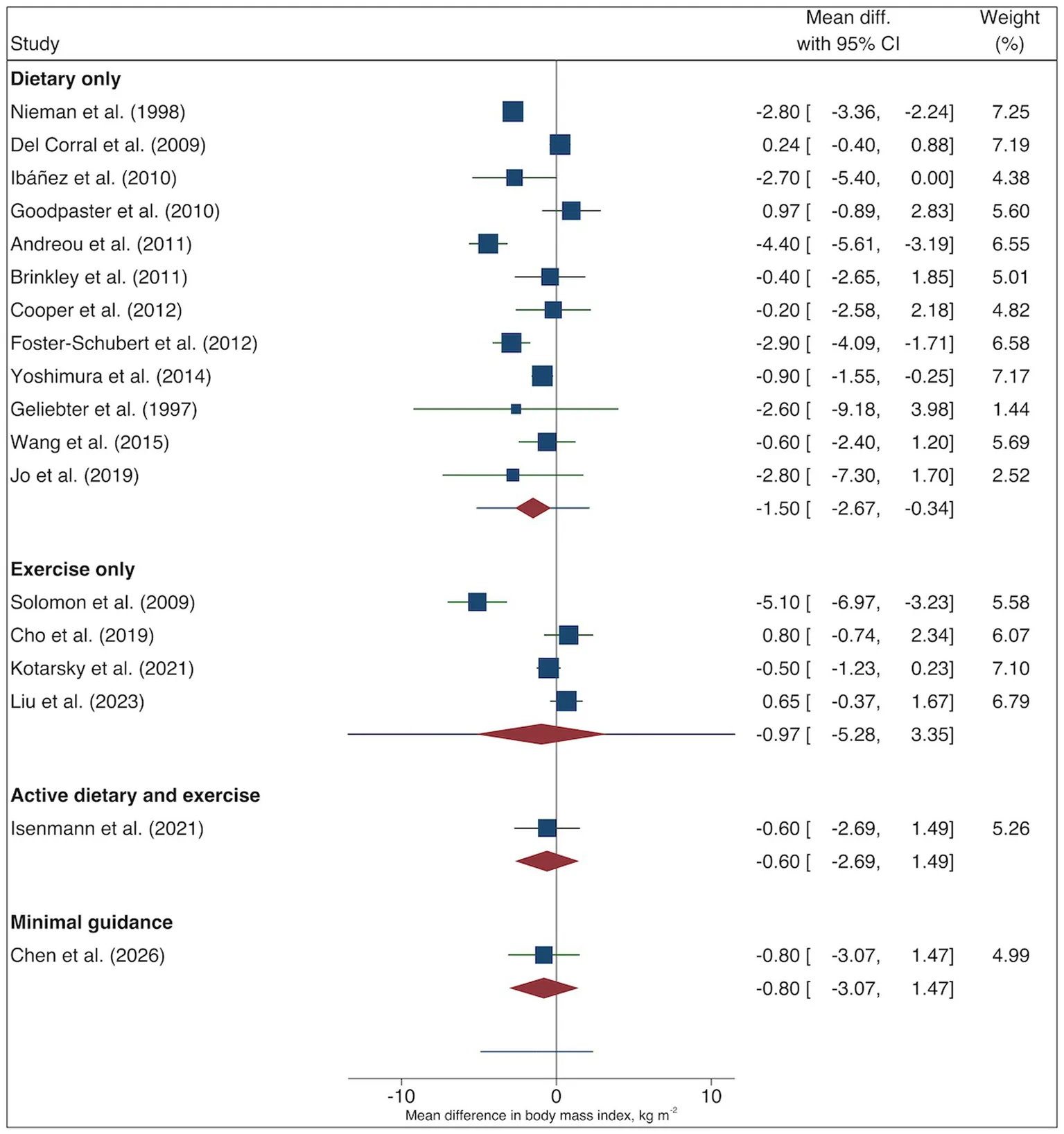
Comparator-stratified random-effects meta-analysis of BMI.

#### 3.3.3 Effect on FFM

When compared against dietary-only controls, 12 reports yielded a mean difference of −0.53 kg (95% confidence interval −2.03 to 0.97), a Tau-squared value of 3.13, an I-squared value of 75.42%, and a prediction interval of −4.75 to 3.69 kg. Two exercise-only comparisons yielded a mean difference of −1.79 kg (−18.49 to 14.92). One active dietary and exercise report yielded a mean difference of −3.80 kg (−10.50 to 2.90). The comparator category did not explain heterogeneity (*p* = 0.480; Figure 4).

**Figure 4:**
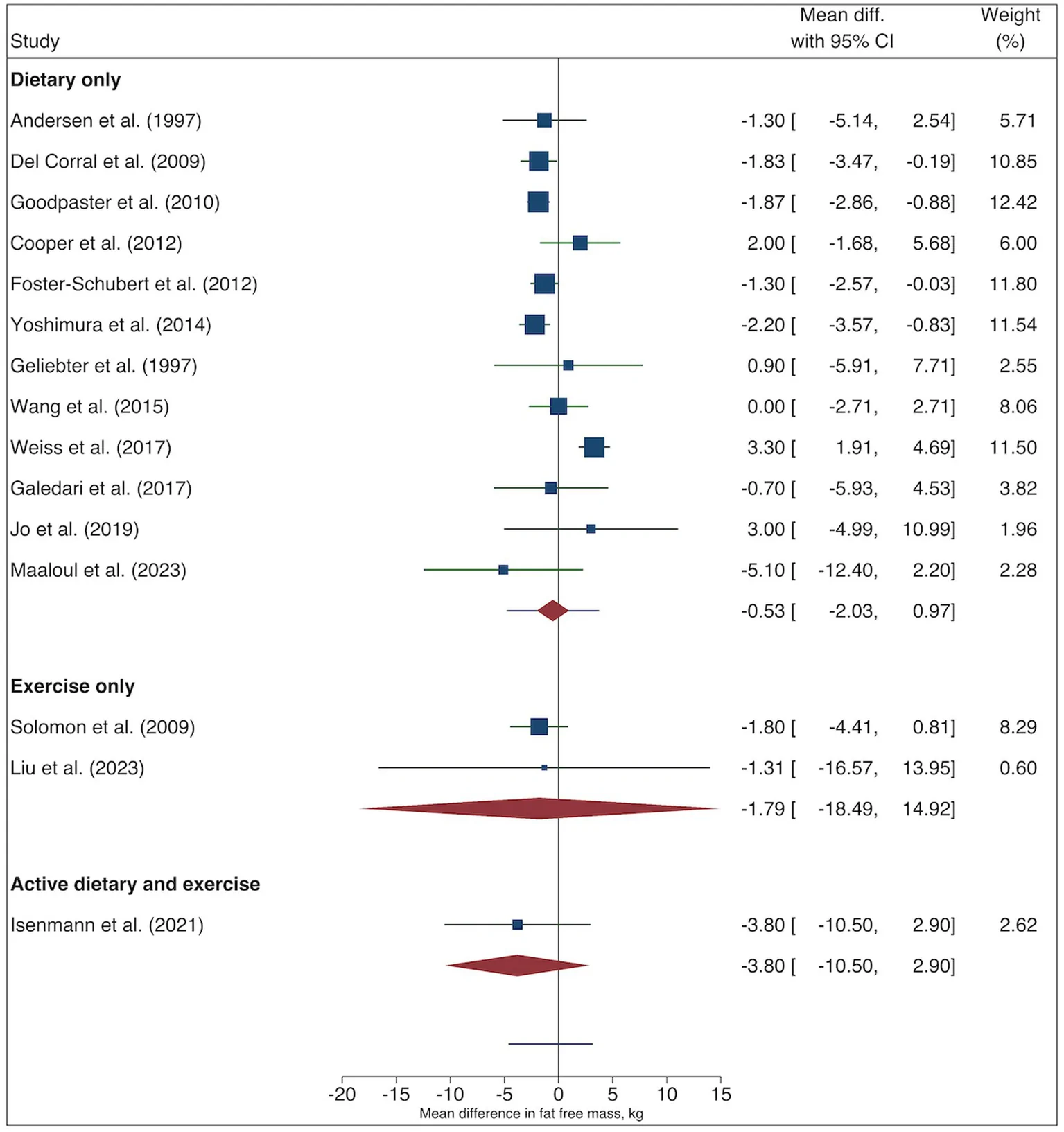
Comparator-stratified random-effects meta-analysis of FFM.

Every FFM confidence interval included the null. Negative mean differences indicate lower FFM in the combined intervention group and do not demonstrate preservation or gain.

#### 3.3.4 Effect on FM%

When compared against dietary-only controls, eight reports yielded a mean difference of −1.40 percentage points (95% confidence interval −4.15 to 1.35), a Tau- squared value of 9.19, an I-squared value of 93.67%, and a prediction interval of −9.34 to 6.55 points. Two exercise-only comparisons yielded a mean difference of 2.02 percentage points (−21.70 to 25.73). One minimal guidance report yielded a mean difference of −1.60 percentage points (−5.84 to 2.64). The comparator category did not explain heterogeneity (*p* = 0.267; Figure 5).

**Figure 5:**
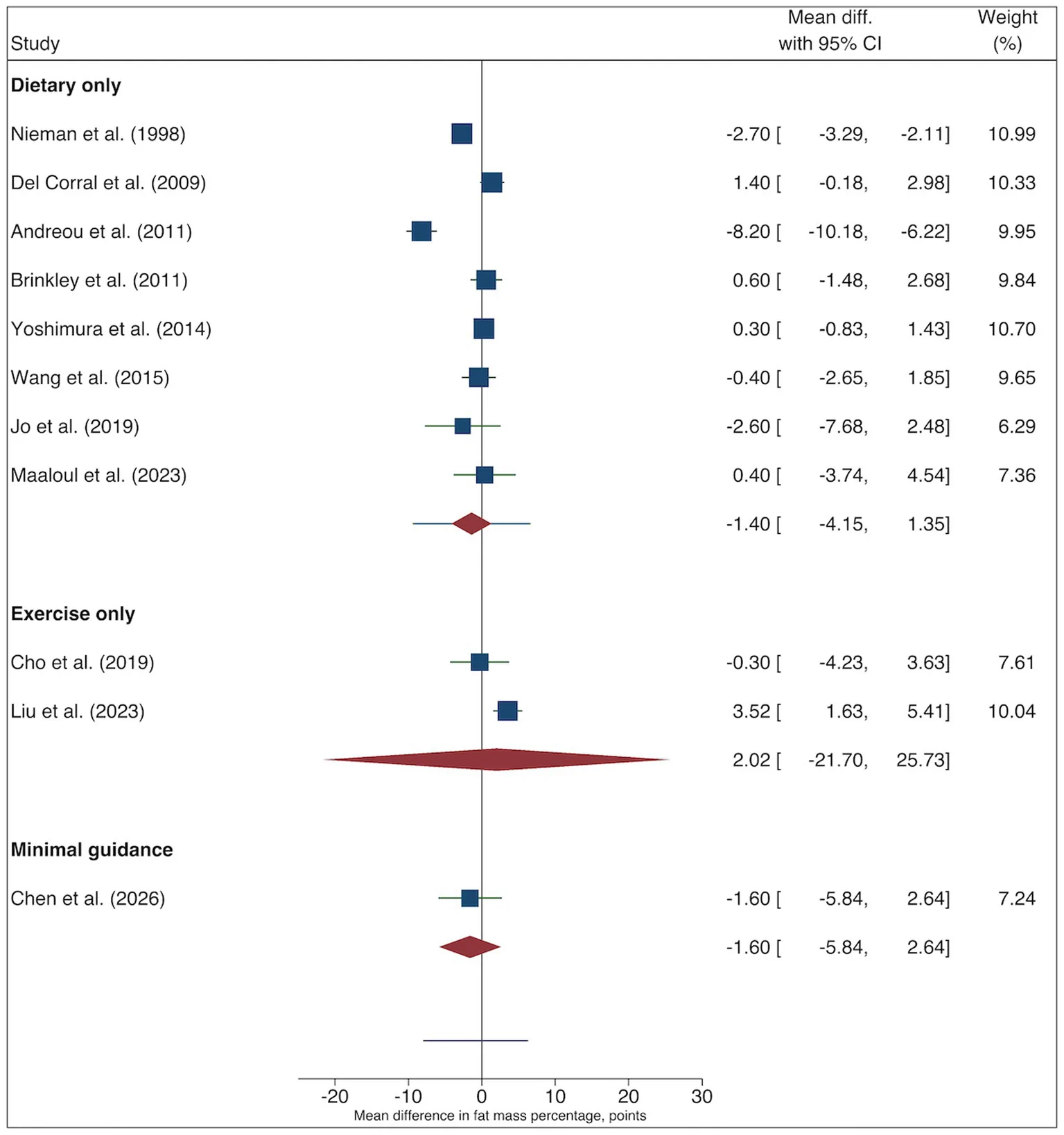
Comparator-stratified random-effects meta-analysis of FM%.

Dietary intervention type, region, duration, and mean age analyses are shown in Supplementary Figures S1 to S5, with numerical moderator results in Supplementary Table S5. These were ecological analyses based on report-level characteristics, often contained few reports, and were not interpreted as direct evidence of differential response. Supplementary Figures S3 and S5 show study-specific estimates without pooled subgroup markers because the relevant strata were sparse.

### 3.4 Sensitivity analyses

The cross-comparator sensitivity model yielded mean differences of −4.13 kg for BW (95% confidence interval −6.76 to −1.50), −1.26 kg m^−2^ for BMI (−2.21 to −0.32), −0.73 kg for FFM (−2.05 to 0.59), and −0.83 points for FM% (−3.04 to 1.37). Every 95% prediction interval crossed the null, so these averages were not interpreted as effects against a common control.

The hierarchical parent-trial dependency analysis retained all 23 reports grouped within 21 unique parent trials and yielded similar estimates: −4.39 kg for BW (−7.02 to −1.76), −1.38 kg m^−2^ for BMI (−2.33 to −0.43), −0.87 kg for FFM (−2.10 to 0.36), and −0.93 points for FM% (−3.04 to 1.19). The report-to-parent trial mapping is provided in Supplementary Table S3. Leave-one-out analyses did not identify a single report that fully accounted for the findings, although heterogeneity remained and inference for FFM and FM% was not robust (Supplementary Figure S6).

### 3.5 Small-study effects and reporting bias

Funnel plots are shown in Supplementary Figure S7. Egger’s tests did not detect asymmetry for BW (*p* = 0.567), BMI (*p* = 0.639), FFM (*p* = 0.966), or FM% (*p* = 0.927). These tests had limited power in small and heterogeneous outcome sets and do not establish the absence of reporting bias.

## 4 Discussion

This review of 23 randomized reports representing 21 unique parent trials found that combined programs were associated with lower BW and BMI when compared with dietary-only controls. Estimates against exercise-only controls were imprecise, while active dietary and exercise and minimal guidance comparisons were represented by single reports. No comparator stratum provided reliable evidence of improved FFM or FM%.

The BW and BMI findings are expressed in natural units, which improves clinical interpretability compared with standardized mean differences (46). However, their prediction intervals crossed the null and extended to effects in the opposite direction. This pattern indicates that the average effect is not a reliable predictor for every future setting (47).

Comparator choice defines the clinical question. A diet-only comparison addresses the incremental contribution of exercise, while an exercise-only comparison addresses the incremental contribution of the dietary prescription. Evidence from adults with overweight or obesity similarly indicates that the added body composition effect of exercise can differ by background diet and population (48, 49).

Statistically significant dietary-only estimates should still be interpreted cautiously. Heterogeneity was high, prediction intervals included no effect, and several older reports incompletely described allocation, deviations, and adherence. The findings support a possible average benefit, not a uniform response or guaranteed clinically important change.

The FFM result does not support preservation. FFM includes skeletal muscle, organs, bone, and body water, and it can vary with hydration and measurement method (50, 51). The mean differences were negative and imprecise. Direct change analyses using consistent methods and adequate reporting are needed before a lean tissue claim is justified.

FM% was especially inconsistent. The dietary-only prediction interval ranged from a substantial reduction to a possible increase, and the exercise-only estimate was extremely imprecise. Differences by mean age or dietary intervention type may reflect ecological confounding factors, comparator distribution, protocol intensity, measurement method, or chance.

Analyses of dietary intervention types cannot establish whether CR with exercise is superior to TRF, alternate-day fasting, or Ramadan fasting with exercise. These intervention types were evaluated in different reports, populations, eras, and comparator conditions. Direct randomized comparisons are required before one combined strategy can be ranked above another.

Clinical importance was not prespecified. Although BW and BMI are now reported in natural units, the review did not define a minimal clinically important difference, and the reported endpoints do not establish improvement in morbidity, function, or quality of life. Weight loss thresholds associated with metabolic benefit vary by outcome and patient context (52).

All 23 reports were retained because companion reports contributed eligible report-specific outcome data. The prespecified hierarchical dependency analysis grouped reports within their 21 unique parent trials. Similar point estimates under that model reduce the concern that the two mapped report pairs alone determined the results. Nevertheless, unknown sampling correlations and incomplete reporting prevent complete resolution of dependence.

Exercise can improve fitness, strength, and weight maintenance even when additional weight loss is modest (53). These benefits should not be conflated with evidence of a body composition advantage, and plausible mechanisms cannot substitute for a precise comparator-specific estimate.

Egger’s tests were non-significant, but asymmetry tests have low power when few heterogeneous reports contribute (54, 55). Selective outcome reporting and unavailable protocols remain plausible explanations, so reporting bias is unresolved rather than excluded.

The main contribution of this review is therefore methodological and clinical clarification. It provides auditable endpoint data, separates randomized comparator questions, uses natural unit effects, displays prediction intervals, and retains distinct dietary intervention types. Earlier direct comparison reviews and fasting syntheses address complementary questions (56, 57).

### 4.1 Strengths and limitations

The strengths of this study include an updated search of four databases, restriction to randomized reports, re-extraction of arm-level endpoints, analysis in natural units, explicit comparator rules, a clear distinction among reports, unique parent trials, and participant observations, restricted maximum likelihood estimation, truncated Hartung–Knapp inference, prediction intervals, RoB 2, GRADE assessment, and the provision of complete Stata code.

Several limitations remain. First, comparator strata were sparse and heterogeneous. Diet, exercise dose, supervision, adherence, population, duration, and measurement method varied within strata. The broad comparator category therefore did not explain the observed inconsistency.

Second, all analyses used postintervention endpoints. Although this avoids mixing change scores with endpoint values, it may be less efficient when baseline measurements are strongly correlated with follow-up values, and random baseline imbalance may influence small trials.

Third, 23 reports represented 21 unique parent trials because two parent trials each contributed companion reports. The hierarchical dependency analysis addressed shared parent trial membership while retaining all report-specific data, but exact within-trial sampling correlations were unavailable. Residual dependence cannot be excluded.

Fourth, older reports often lacked complete details on allocation concealment, adherence, prespecified analyses, and body composition procedures. RoB 2 judgments therefore frequently remained at some concerns, and intervention detail was recorded as not reported when it could not be verified.

Fifth, study-level subgroup and meta-regression analyses had limited power and were vulnerable to ecological confounding. Sixth, funnel plots and Egger’s tests could not exclude reporting bias. Finally, no minimal clinically important difference was prespecified.

Future trials should use clearly defined comparators, report endpoint and change data in natural units, publish protocols and analysis plans, standardize body composition methods, document adherence, and directly compare dietary intervention types within a common exercise program.

## 5 Conclusion

Combined exercise-diet interventions may reduce BW and BMI relative to the comparator conditions used in randomized trials, but high heterogeneity and prediction intervals crossing the null limit confidence in the effect expected in a new setting. Comparisons with exercise-only controls were imprecise.

The evidence does not establish FFM preservation, a reliable reduction in FM%, the superiority of one dietary intervention type, or clinical importance. Comparator-specific, adequately powered trials with transparent reporting in natural units are required.

## Data Availability

The original contributions presented in the study are included in the article/Supplementary material, further inquiries can be directed to the corresponding author.
