# Supplementary material for: Combined exercise and dietary interventions for body composition in adults with overweight or obesity: a comparator-stratified systematic review and meta-analysis of randomized trials
Source: Front Nutr. 2026 Oct 28;13:1969222. doi: 10.3389/fnut.2026.1969222 (PMC13751346; doi:10.3389/fnut.2026.1969222)
Supplement: Supplementary file 1 [file Supplementary_file_1.docx]

# Combined Exercise and Dietary Interventions for Body Composition in Adults with Overweight or Obesity: A Comparator Stratified Systematic Review and Meta-Analysis of Randomized Trials

# Supplementary Table S1. Search strategies and archived record counts

| **Database** | **Coverage** | **Executable reconstruction of documented concepts** | **Archived count** |
| --- | --- | --- | --- |
| PubMed | Inception-September 28th, 2026 | ((((((("Overweight"[Mesh]) OR ("Obesity"[Mesh])) OR (obes*[Title/Abstract])) OR (overweight*[Title/Abstract])) OR (excess weight[Title/Abstract])) AND ((((((((((((((((("Exercise Therapy"[Mesh]) OR ("Exercise"[Mesh])) OR (exercis*[Title/Abstract])) OR (physical activity[Title/Abstract])) OR (physical activities[Title/Abstract])) OR (resistance training[Title/Abstract])) OR (strength training[Title/Abstract])) OR (weight training[Title/Abstract])) OR (aerobic exercise[Title/Abstract])) OR (aerobic training[Title/Abstract])) OR (endurance training[Title/Abstract])) OR (interval training[Title/Abstract])) OR (high intensity interval training[Title/Abstract])) OR (high-intensity interval training[Title/Abstract])) OR (HIIT[Title/Abstract])) OR (concurrent training[Title/Abstract])) OR (walking[Title/Abstract]))) AND ((((((((("Diet Therapy"[Mesh]) OR ("Caloric Restriction"[Mesh])) OR (Fasting[Title/Abstract])) OR (Intermittent Fasting[Title/Abstract])) OR (dietary intervention[Title/Abstract])) OR (dietary interventions[Title/Abstract])) OR (diet intervention[Title/Abstract])) OR (dietary program[Title/Abstract])) OR (weight loss diet[Title/Abstract]))) AND (((((((("Randomized Controlled Trial" [Publication Type]) OR (Controlled Clinical Trial[Title/Abstract])) OR (randomized controlled trial[Title/Abstract])) OR (randomized controlled trials[Title/Abstract])) OR (randomised controlled trial[Title/Abstract])) OR (randomised controlled trials[Title/Abstract])) OR (RCT[Title/Abstract])) OR (RCTs[Title/Abstract])) | 2,323 |
| Embase | Inception-September 28th, 2026 | ('obesity'/exp OR 'overweight'/exp OR obes*:ti,ab,kw OR overweight*:ti,ab,kw OR 'excess weight':ti,ab,kw) AND ('exercise'/exp OR 'exercise therapy'/exp OR 'physical activity'/exp OR exercis*:ti,ab,kw OR 'physical activity':ti,ab,kw OR 'resistance training':ti,ab,kw OR 'strength training':ti,ab,kw OR 'weight training':ti,ab,kw OR 'aerobic exercise':ti,ab,kw OR 'aerobic training':ti,ab,kw OR 'endurance training':ti,ab,kw OR 'interval training':ti,ab,kw OR 'high intensity interval training':ti,ab,kw OR hiit:ti,ab,kw OR 'concurrent training':ti,ab,kw OR walking:ti,ab,kw) AND ('diet therapy'/exp OR 'caloric restriction'/exp OR 'fasting'/exp OR 'intermittent fasting'/exp OR 'dietary intervention':ti,ab,kw OR 'dietary interventions':ti,ab,kw OR 'diet intervention':ti,ab,kw OR 'dietary program':ti,ab,kw OR 'weight loss diet':ti,ab,kw) AND ('randomized controlled trial'/exp OR 'controlled clinical trial'/de OR 'randomization'/de OR 'randomized controlled trial':ti,ab,kw OR 'randomized controlled trials':ti,ab,kw OR 'randomised controlled trial':ti,ab,kw OR 'randomised controlled trials':ti,ab,kw OR rct:ti,ab,kw OR rcts:ti,ab,kw) | 5,648 |
| Cochrane Library/CENTRAL | Inception-September 28th, 2026 | ((MeSH descriptor: [Obesity] explode all trees) OR (MeSH descriptor: [Overweight] explode all trees) OR ((obes* OR overweight* OR "excess weight"):ti,ab,kw)) AND ((MeSH descriptor: [Exercise] explode all trees) OR ((exercis* OR "physical activity" OR "physical activities" OR "resistance training" OR "strength training" OR "weight training" OR "aerobic exercise" OR "aerobic training" OR "endurance training" OR "interval training" OR "high intensity interval training" OR "high-intensity interval training" OR HIIT OR "concurrent training" OR walking):ti,ab,kw)) AND ((MeSH descriptor: [Diet Therapy] explode all trees) OR (MeSH descriptor: [Caloric Restriction] explode all trees) OR (("dietary intervention" OR "dietary interventions" OR "diet intervention" OR "dietary program" OR "weight loss diet" OR "caloric restriction" OR "calorie restriction" OR "energy restriction" OR "energy deficit" OR hypocalor* OR "low calorie diet" OR "very low calorie diet" OR "meal replacement" OR "intermittent fasting" OR "alternate day fasting" OR "alternate-day fasting" OR "time restricted eating" OR "time-restricted eating" OR "time restricted feeding" OR "time-restricted feeding" OR TRE OR TRF OR "5:2 fasting" OR "5 2 fasting"):ti,ab,kw)) | 3,592 |
| Web of Science Core Collection | Inception-September 28th, 2026 | TS=((obes* OR overweight* OR "excess weight") AND ( exercis* OR (physical NEAR/1 activit*) OR (resistance NEAR/1 train*) OR (strength NEAR/1 train*) OR (weight NEAR/1 train*) OR (aerobic NEAR/1 (exercis* OR train*)) OR (endurance NEAR/1 train*) OR (interval NEAR/1 train*) OR "high intensity interval training" OR HIIT OR (concurrent NEAR/1 train*) OR walking) AND (( (diet* OR nutrition*) NEAR/3 ( intervention* OR program* OR treatment* OR therap* OR restrict* OR hypocalor* OR "weight loss" )) OR (calori* NEAR/1 restrict*) OR (energy NEAR/1 restrict*) OR (energy NEAR/1 deficit*) OR hypocalor* OR "low calorie diet" OR "very low calorie diet" OR "weight loss diet" OR (meal NEAR/1 replacement*) OR "intermittent fasting" OR "alternate day fasting" OR "alternate-day fasting" OR ((time NEAR/1 restrict*) NEAR/2 (eat* OR feed*)) OR "time restricted eating" OR "time-restricted eating" OR "time restricted feeding" OR "time-restricted feeding" OR TRE OR TRF OR "5:2 fasting" OR ketogenic OR "low carbohydrate diet" OR "low-carbohydrate diet" OR "Ramadan fasting" ) AND ( "randomized controlled trial" OR "randomized controlled trials" OR "randomised controlled trial" OR "randomised controlled trials" OR RCT OR RCTs OR randomi* OR "randomly assigned" OR "randomly allocated" OR (random* NEAR/3 (assign* OR allocat*)) OR ((controlled OR clinical) NEAR/1 trial*) OR (parallel NEAR/1 group*) OR crossover OR "cross over" OR ((single OR double) NEAR/1 (blind* OR mask*)) OR placebo*) | 4,965 |

Note: Counts reproduce the archived searches conducted through September 28, 2026. CENTRAL is restricted to controlled trial records by database design.

# Supplementary Table S2. Study level and outcome specific RoB 2 judgments

| **Report** | **Outcomes** | **D1** | **D2** | **D3** | **D4 BW and BMI** | **D4 FFM and FM%** | **D5** | **Overall** | **Rationale** |
| --- | --- | --- | --- | --- | --- | --- | --- | --- | --- |
| Andersen et al. (1997) | BW, FFM | Some concerns | Some concerns | Some concerns | Low risk | Some concerns | Some concerns | Some concerns | Allocation concealment, attrition detail, or prespecified analysis information was incompletely reported. Anthropometric outcomes were objective; body composition measures remained method dependent. |
| Nieman et al. (1998) | BW, BMI, FM% | Some concerns | Some concerns | Some concerns | Low risk | Some concerns | Some concerns | Some concerns | Allocation concealment, attrition detail, or prespecified analysis information was incompletely reported. Anthropometric outcomes were objective; body composition measures remained method dependent. |
| Del Corral et al. (2009) | BW, BMI, FFM, FM% | Some concerns | Some concerns | Some concerns | Low risk | Some concerns | Some concerns | Some concerns | Allocation concealment, attrition detail, or prespecified analysis information was incompletely reported. Anthropometric outcomes were objective; body composition measures remained method dependent. |
| Solomon et al. (2009) | BW, BMI, FFM | Some concerns | Some concerns | Some concerns | Low risk | Some concerns | Some concerns | Some concerns | Allocation concealment, attrition detail, or prespecified analysis information was incompletely reported. Anthropometric outcomes were objective; body composition measures remained method dependent. |
| Ibáñez et al. (2010) | BW, BMI | Some concerns | Some concerns | Some concerns | Low risk | Some concerns | Some concerns | Some concerns | Allocation concealment, attrition detail, or prespecified analysis information was incompletely reported. Anthropometric outcomes were objective; body composition measures remained method dependent. |
| Goodpaster et al. (2010) | BW, BMI, FFM | Some concerns | Some concerns | Some concerns | Low risk | Some concerns | Some concerns | Some concerns | Allocation concealment, attrition detail, or prespecified analysis information was incompletely reported. Anthropometric outcomes were objective; body composition measures remained method dependent. |
| Andreou et al. (2011) | BW, BMI, FM% | Some concerns | Some concerns | Some concerns | Low risk | Some concerns | Some concerns | Some concerns | Allocation concealment, attrition detail, or prespecified analysis information was incompletely reported. Anthropometric outcomes were objective; body composition measures remained method dependent. |
| Brinkley et al. (2011) | BW, BMI, FM% | Some concerns | Some concerns | Some concerns | Low risk | Some concerns | Some concerns | Some concerns | Allocation concealment, attrition detail, or prespecified analysis information was incompletely reported. Anthropometric outcomes were objective; body composition measures remained method dependent. |
| Cooper et al. (2012) | BW, BMI, FFM | Some concerns | Some concerns | Some concerns | Low risk | Some concerns | Some concerns | Some concerns | Allocation concealment, attrition detail, or prespecified analysis information was incompletely reported. Anthropometric outcomes were objective; body composition measures remained method dependent. |
| Foster-Schubert et al. (2012) | BW, BMI, FFM | Some concerns | Some concerns | Some concerns | Low risk | Some concerns | Some concerns | Some concerns | Allocation concealment, attrition detail, or prespecified analysis information was incompletely reported. Anthropometric outcomes were objective; body composition measures remained method dependent. |
| Yoshimura et al. (2014) | BW, BMI, FFM, FM% | Some concerns | Some concerns | Some concerns | Low risk | Some concerns | Some concerns | Some concerns | Allocation concealment, attrition detail, or prespecified analysis information was incompletely reported. Anthropometric outcomes were objective; body composition measures remained method dependent. |
| Geliebter et al. (1997) | BW, BMI, FFM | Some concerns | Some concerns | Some concerns | Low risk | Some concerns | Some concerns | Some concerns | Allocation concealment, attrition detail, or prespecified analysis information was incompletely reported. Anthropometric outcomes were objective; body composition measures remained method dependent. |
| Wang et al. (2015) | BW, BMI, FFM, FM% | Some concerns | Some concerns | Some concerns | Low risk | Some concerns | Some concerns | Some concerns | Allocation concealment, attrition detail, or prespecified analysis information was incompletely reported. Anthropometric outcomes were objective; body composition measures remained method dependent. |
| Weiss et al. (2017) | BW, FFM | Some concerns | Some concerns | Some concerns | Low risk | Some concerns | Some concerns | Some concerns | Allocation concealment, attrition detail, or prespecified analysis information was incompletely reported. Anthropometric outcomes were objective; body composition measures remained method dependent. |
| Galedari et al. (2017) | BW, FFM | Some concerns | Some concerns | Some concerns | Low risk | Some concerns | Some concerns | Some concerns | Allocation concealment, attrition detail, or prespecified analysis information was incompletely reported. Anthropometric outcomes were objective; body composition measures remained method dependent. |
| Cho et al. (2019) | BW, BMI, FM% | Some concerns | Some concerns | Some concerns | Low risk | Some concerns | Some concerns | Some concerns | Allocation concealment, attrition detail, or prespecified analysis information was incompletely reported. Anthropometric outcomes were objective; body composition measures remained method dependent. |
| Jo et al. (2019) | BW, BMI, FFM, FM% | Some concerns | Some concerns | Some concerns | Low risk | Some concerns | Some concerns | Some concerns | Allocation concealment, attrition detail, or prespecified analysis information was incompletely reported. Anthropometric outcomes were objective; body composition measures remained method dependent. |
| Isenmann et al. (2021) | BW, BMI, FFM | Some concerns | Some concerns | Some concerns | Low risk | Some concerns | Some concerns | Some concerns | Allocation concealment, attrition detail, or prespecified analysis information was incompletely reported. Anthropometric outcomes were objective; body composition measures remained method dependent. |
| Kotarsky et al. (2021) | BW, BMI | Some concerns | Some concerns | Some concerns | Low risk | Some concerns | Some concerns | Some concerns | Allocation concealment, attrition detail, or prespecified analysis information was incompletely reported. Anthropometric outcomes were objective; body composition measures remained method dependent. |
| Haganes et al. (2022) | BW | Some concerns | Some concerns | Some concerns | Low risk | Some concerns | Some concerns | Some concerns | Allocation concealment, attrition detail, or prespecified analysis information was incompletely reported. Anthropometric outcomes were objective; body composition measures remained method dependent. |
| Liu et al. (2023) | BW, BMI, FFM, FM% | Some concerns | Some concerns | Some concerns | Low risk | Some concerns | Some concerns | Some concerns | Allocation concealment, attrition detail, or prespecified analysis information was incompletely reported. Anthropometric outcomes were objective; body composition measures remained method dependent. |
| Maaloul et al. (2023) | BW, FFM, FM% | Some concerns | Some concerns | Some concerns | Low risk | Some concerns | Some concerns | Some concerns | Allocation concealment, attrition detail, or prespecified analysis information was incompletely reported. Anthropometric outcomes were objective; body composition measures remained method dependent. |
| Chen et al. (2026) | BW, BMI, FM% | Some concerns | Some concerns | Some concerns | Low risk | Some concerns | Some concerns | Some concerns | Allocation concealment, attrition detail, or prespecified analysis information was incompletely reported. Anthropometric outcomes were objective; body composition measures remained method dependent. |

D1, randomization process; D2, deviations from intended interventions; D3, missing outcome data; D4, outcome measurement; D5, selection of the reported result. Judgments concern the effect of assignment to intervention. Some concerns was used when reporting did not resolve a signaling question.

# Supplementary Table S3. Study characteristics, intervention details, comparator verification, and outcomes

| **Study** | **Region** | **Population** | **Age** | **Sample size (N)** | **Intervention description** | **Duration** | **Actual comparator** | **Outcomes** | **Parent trial** |
| --- | --- | --- | --- | --- | --- | --- | --- | --- | --- |
| Andersen et al (1997) | USA | Healthy obese women, 38years old | 38.1±7.6 | I: 11  C: 8 | Diet (CR) plus resistance training with calisthenics and used Nautilus (Independence, VA) and Cybex (Ronkonkoma NY) gym equipment. | 24w | Diet only | BW, FM, FFM | Andersen et al (1997) |
| Nieman et al (1998) | USA | Obese women, 45 years old | 38 | I: 22  C: 22 | Reducing diet (CR) and exercise training with walk and indoor track | 12W | Diet only | BW, BMI, FM% | Nieman et al (1998) |
| Del Corral et al (2009) | USA | Healthy overweight women, 34 years old | 35.2 ±7.0 | I: 46  C: 34 | Diet-resistance exercise (DR) | 22W | Diet only | BW, BMI, FM%, FM, FFM | Del Corral et al (2009) |
| Solomon et al (2009) | USA | Obese elderly people, 66 years old | 66 ±1 | I: 8  C: 8 | Aerobic exercise training and dietary counseling | 12W | Eucaloric or hypocaloric | BW, BMI, FM, FFM | Solomon et al (2009) |
| Ibáñez et al (2010) | Spain | Obese women, 50 years old | 48.6 ± 6.4 | I: 13  C: 9 | Diet-resistance exercise (DR) | 16W | Diet only | BW, BMI, FFM | Ibáñez et al (2010) |
| Goodpaster et al (2010) | USA | Severely obese adults, 46 years old | 47.5 ±6.2 | I: 63  C: 67 | physical activity in a weekly diary | 6M | Delayed-PE | BW, BMI, FM, FFM | Goodpaster parent trial |
| Andreou et al (2011) | Cyprus | Healthy obese women，33years old | 35±11.2 | I: 111  C: 95 | diet and exercise | 18w | Diet only | BW, BMI, FM% | Andreou et al (2011) |
| Brinkley et al (2011) | USA | Overweight and obese women, 58 years old | 58.5±5.3 | I: 22  C: 22 | diet and exercise | 20W | Caloric restriction | BW, BMI, FM%, FM | NCT00664729 |
| Cooper et al (2012) | USA | Severely obese adults, 33 years old | 46.8 ±6.5 | I: 45  C: 45 | diet and exercise | 6M | Diet only | BW, BMI, FM, FFM | Goodpaster parent trial |
| Foster-Schubert et al (2012) | USA | Overweight to obese women, 58 years old | 58.0 ±4.5 | I: 117  C: 87 | diet and exercise | 12M | Diet only | BW, BMI, FM%, FM, FFM | Foster-Schubert et al (2012) |
| Yoshimura et al (2014) | Japan | Adults with obesity, 55years old | 61 ± 2 | I: 15  C: 18 | diet and exercise | 12W | Caloric restriction | BW, BMI, FM%, FM, FFM | Yoshimura et al (2014) |
| Geliebter et al (1997) | USA | Overweight and obese people, 35 years old | 35 ± 6 | I: 23  C: 22 | diet and exercise | 8W | Diet only | BW, BMI, FM, FFM | Geliebter et al (1997) |
| Wang et al (2015) | USA | Overweight or obese women, 58 years old | 58.4±5.2 | I: 48  C: 22 | diet and exercise | 20W | Caloric restriction | BW, BMI, FM%, FM, FFM | NCT00664729 |
| Weiss et al (2017) | USA | Overweight women and men, 57 years old | 57±7 | I: 19  C: 17 | diet and exercise | 17W | Caloric restriction | BW, FM, FFM | Weiss et al (2017) |
| Galedari et al (2017) | Iran | Untrained overweight male, 30 years old | 30.8 ± 7.6 | I: 10  C: 8 | diet and exercise | 12W | Caloric restriction | BW, FM, FFM | Galedari et al (2017) |
| Cho et al (2019) | Korea | Overweight or obese adults, 35 years old | 34.5 ± 5.7 | I: 9  C: 5 | diet and exercise | 8W | Exercise only | BW, BMI, FM%, FM | Cho et al (2019) |
| Jo et al (2019) | USA | Obese people, 58 years old | 58 ± 3 | I: 6  C: 5 | diet and exercise | 12W | Resistance training | BW, BMI, FM%, FM, FFM | Jo et al (2019) |
| Isenmann et al (2021) | Germany | Fitness enthusiasts with a BMI greater than 25, 27 years old | 27.9 ± 5.3 | I: 18  C: 17 | diet and exercise | 14W | Macronutrient-Based Diet | BW, BMI, FM, FFM | Isenmann et al (2021) |
| Kotarsky et al (2021) | USA | Overweight and obese adults, 44 years old | 45 ± 3 | I: 11  C: 10 | diet and exercise | 8W | Normal eating | BW, BMI, FM | Kotarsky et al (2021) |
| Haganes et al (2022) | Norway | Overweight to obese women, 36 years old | 37.3±5.7 | I: 32  C: 33 | diet and exercise | 7W | Exercise only | BW, FM | Haganes et al (2022) |
| Liu et al (2023) | China | Female college students suffering from hidden obesity, 20 years old | 19.93 ± 0.61 | I: 19  C: 19 | diet and exercise | 8W | Exercise only | BW, BMI, FM%, FM, FFM | Liu et al (2023) |
| Maaloul et al (2023) | Tunisia | Obese men, 32 years old | 30.2 ± 6.1 | I: 10  C: 10 | diet and exercise | 4W | Usual lifestyle | BW, FM%, FM, FFM | Maaloul et al (2023) |
| Chen et al (2026) | China | Obese young adults, 18–25 years | 20.4 ± 1.6 | I: 28  C: 14 | diet and exercise | 8W | Chinese Dietary Guidelines | BW, BMI, FM% | Chen et al (2026) |

Note: The parent trial variable was used in the hierarchical sensitivity model.

# Supplementary Table S4. GRADE certainty assessment

| **Outcome and comparator** | **Reports** | **Risk of bias** | **Inconsistency** | **Indirectness** | **Imprecision** | **Reporting bias** | **Certainty** | **Explanation** |
| --- | --- | --- | --- | --- | --- | --- | --- | --- |
| BW, dietary only | 16 | Serious | Very serious | Not serious | Serious | Undetected but uncertain | Very low | Incomplete reporting; high heterogeneity; prediction interval crossed the null. |
| BMI, dietary only | 12 | Serious | Very serious | Not serious | Serious | Undetected but uncertain | Very low | Incomplete reporting; high heterogeneity; prediction interval crossed the null. |
| FFM, dietary only | 12 | Serious | Serious | Serious | Serious | Undetected but uncertain | Very low | Measurement methods varied; confidence and prediction intervals crossed the null. |
| FM percentage, dietary only | 8 | Serious | Very serious | Serious | Very serious | Not assessable | Very low | Very high heterogeneity, sparse evidence, and wide intervals. |
| All exercise only contrasts | 2 to 5 | Serious | Serious | Not serious | Very serious | Not assessable | Very low | Few reports and very wide confidence intervals. |
| Active dietary and exercise or minimal guidance | 1 each | Serious | Not assessable | Not serious | Very serious | Not assessable | Very low | Single report evidence with imprecise estimates. |

# Supplementary Table S5. Exploratory moderator analyses

| **outcome** | **moderator** | **k** | **p_moderator** | **tau2 resid** | **r2** |
| --- | --- | --- | --- | --- | --- |
| BW | Comparator category | 23 | 0.90584731 |  |  |
| BW | Comparator meta regression | 23 | 0.95116448 | 27.411915 | 0 |
| BW | Duration in weeks | 23 | 0.238617 | 23.327127 | 2.6064801 |
| BW | Mean study age | 23 | 0.96978325 | 25.451347 | 0 |
| BMI | Comparator category | 18 | 0.85135841 |  |  |
| BMI | Comparator meta regression | 18 | 0.92959231 | 3.2277489 | 0 |
| BMI | Duration in weeks | 18 | 0.5353986 | 2.7880547 | 0 |
| BMI | Mean study age | 18 | 0.17920138 | 2.520309 | 6.3753781 |
| FFM | Comparator category | 15 | 0.47952002 |  |  |
| FFM | Comparator meta regression | 15 | 0.6292209 | 3.0744379 | 0 |
| FFM | Duration in weeks | 15 | 0.94359922 | 3.1903594 | 0 |
| FFM | Mean study age | 15 | 0.44636726 | 2.9742944 | 0 |
| FM% | Comparator category | 11 | 0.26660734 |  |  |
| FM% | Comparator meta regression | 11 | 0.47863007 | 8.7548361 | 0.42754492 |
| FM% | Duration in weeks | 11 | 0.61461174 | 9.5006676 | 0 |
| FM% | Mean study age | 11 | 0.89999866 | 9.8238363 | 0 |

Note: Moderator analyses were exploratory and study level. They were not interpreted as randomized comparisons.

**Supplementary Figure S1.** Exploratory BW subgroup analysis by dietary intervention type.

**Supplementary Figure S2.** Exploratory BMI subgroup analysis by dietary intervention type.

**Supplementary Figure S3.** Exploratory fat free mass estimates stratified by dietary intervention type. Pooled subgroup markers are omitted because several strata contained fewer than three reports.

# Supplementary Figure S4. Exploratory FFM subgroup analysis by geographical region.

**Supplementary Figure S5.** Exploratory fat mass percentage estimates stratified by mean study age. Pooled subgroup markers are omitted because the younger stratum contained two reports.

**Supplementary Figure S6.** Leave one out analyses (Panel a).

**Supplementary Figure S6.** Leave one out analyses (Panel b).

**Supplementary Figure S6.** Leave one out analyses (Panel c).

**Supplementary Figure S6**. Leave one out analyses (Panel d).

**Supplementary Figure S7.** Funnel plots.
